# Personality-related learning pathways in virtual simulation: a theory-informed mixed-methods study among standardized training nurses

**DOI:** 10.3389/fpsyg.2026.1876039

**Published:** 2026-08-12

**Authors:** Zhonglei Zhao, Xixi Li, Tong Zhou, Luyao Yang, Yunxia Li, Bo Yang

**Affiliations:** 1 North Sichuan Medical College, Nanchong, China; 2 Suining Central Hospital, Suining, China; 3 Zigong Fourth People’s Hospital, Zigong, China; 4 People’s Hospital of Leshan, Leshan, China

**Keywords:** learning outcomes, mixed-methods study, personality traits, triadic reciprocal determinism, virtual simulation

## Abstract

**Objective:**

To examine the associations among personality traits, learning behaviors, and learning outcomes in virtual simulation training among standardized training nurses.

**Methods:**

An explanatory sequential mixed-methods design was used. In the quantitative phase, 128 nurses completed a virtual simulation program on geriatric fall management. Knowledge was assessed before and after training, personality was measured using the Big Five Inventory-2, and learning behaviors were obtained from the platform. Hierarchical regression was performed with post-test knowledge as the outcome. In the qualitative phase, 17 participants underwent interviews using the Zaltman Metaphor Elicitation Technique.

**Results:**

Post-test knowledge scores were significantly higher than pre-test scores (78.44 ± 7.28 vs. 70.93 ± 7.21, *p* < 0.001). The final regression model explained 57.3% of the variance in post-test knowledge. Baseline knowledge, number of learning sessions (*β* = 0.30, *p* < 0.001), and average simulation scores (*β* = 0.25, *p* = 0.001) remained significant, whereas the five personality dimensions did not. Qualitative findings identified five constructs: learning support, knowledge construction, emotional experience, learning motivation, and external learning pressure. Conscientiousness was associated with planning and repetition, Neuroticism with anxiety and time pressure, and Agreeableness with peer support.

**Conclusion:**

Virtual simulation training was associated with improved fall-management knowledge. Learning behaviors were more proximal correlates of knowledge outcomes than personality traits, while personality differences may be reflected in distinct behavioral, emotional, and interpersonal patterns of engagement.

## 1 Introduction

The widespread adoption of digital learning technologies has foregrounded a persistent question in education: why do learners who are provided with the same instructional resources often achieve markedly different learning outcomes? Individual differences are increasingly recognized as important in understanding such variation, particularly because learners differ in how they regulate their learning, process information, respond emotionally to challenges, and engage with digital environments (Tokiwa, 2025; Chai and Ye, 2024). As educational technologies evolve from static online content toward more interactive and immersive forms of learning, understanding how dispositional characteristics relate to learners’ behaviors and experiences has become increasingly important.

Among individual difference variables, the Big Five personality traits have been consistently associated with academic performance and learning-related behaviors. Conscientiousness, in particular, has been linked to stronger achievement through goal-directed self-discipline, sustained effort, planning, and strategic regulation of learning. By contrast, Neuroticism has often been associated with poorer performance in evaluative or unfamiliar learning situations because heightened anxiety and maladaptive emotional responses may compete with task-relevant cognitive resources (Chen et al., 2025; Apostolov and Geldenhuys, 2022). These findings suggest that personality traits may be relevant not only to how much effort learners invest, but also to how they regulate their behavior and respond emotionally to learning demands.

Virtual simulation provides a particularly informative context in which to examine these differences. It is not merely another form of online content delivery. Learners are often required to regulate when and how frequently they practice, monitor their performance, interpret digital feedback, tolerate errors and uncertainty, and repeatedly adjust their actions within an evaluative environment. These features make self-regulation and emotional responses especially salient. Learners may differ not only in the amount of practice they undertake, but also in how they respond to feedback, perceived performance pressure, and opportunities for repeated practice. Platform-recorded behavioral indicators, such as the frequency of learning sessions and simulation performance, can further provide observable traces of how learners engage with the learning environment. Personality traits may therefore be particularly relevant in virtual simulation because they are associated with differences in persistence, effort regulation, anxiety-related responses, and interpersonal engagement.

At the same time, virtual simulation has distinctive environmental characteristics. Its high contextual fidelity enables learners to engage with clinically realistic situations, while the absence of direct risk to real patients creates opportunities for repeated practice and error correction. This combination of immersion, feedback, repetition, and reduced real-world clinical risk may support learning, but it may also be experienced differently by learners with different dispositional characteristics. For example, a learner with stronger self-regulatory tendencies may use repeated attempts to consolidate knowledge, whereas a learner who is more sensitive to evaluation and uncertainty may experience the same scoring or feedback system as a source of pressure. Virtual simulation therefore offers a useful setting for examining how personal characteristics, learning behaviors, and environmental conditions may jointly relate to learning outcomes.

To conceptualize these relationships, the present study adopts Bandura’s triadic reciprocal determinism as its overarching theoretical framework (Bandura, 1986). The theory proposes that personal factors, behaviors, and environmental conditions operate as interrelated and mutually influential components rather than as isolated determinants (Wu, 2017). Applied to virtual simulation, this perspective suggests that learning outcomes should not be understood solely as the product of personality, learner effort, or platform design. Instead, learners bring dispositional characteristics into the simulation environment, engage in different behavioral patterns, respond cognitively and emotionally to environmental features, and may in turn contribute to the social and learning environment through their own actions. This framework is therefore well suited to integrating personality characteristics, platform-recorded learning behaviors, and learners’ subjective experiences within a single explanatory structure.

Virtual simulation is also broadly consistent with the principles of experiential learning. Learners encounter simulated clinical situations, receive feedback on their performance, reconsider their understanding, and apply revised knowledge in subsequent attempts. Kolb’s experiential learning cycle provides useful background for understanding why repeated simulation may support learning through experience, reflection, conceptual development, and further action (Kolb, 2015; Alruwaili et al., 2025). In the present study, however, Kolb’s model was not used as a separate analytical framework or as an *a priori* structure for qualitative coding. The study design, mixed-methods integration, and interpretation of the relationships among personality characteristics, learning behaviors, and the simulation environment were guided primarily by Bandura’s triadic reciprocal determinism.

The present study focuses on Chinese Standardized Training Nurses, a group in a transitional stage between formal nursing education and increasingly independent clinical practice. In China, newly graduated nurses are required to complete a mandatory, hospital-based standardized training program before practicing independently (Health and Family Planning Commission of Sichuan Province, 2018). During this period, nurses rotate across clinical departments, acquire core competencies under supervision, and simultaneously adapt theoretical knowledge to clinical situations. These dual demands of learning and performing may make individual differences in self-regulation, stress reactivity, and emotional responses particularly relevant. This may be especially important in high-stakes clinical situations such as geriatric falls, in which Standardized Training Nurses may have limited experience in rapid assessment, emergency decision-making, and subsequent management (Yang et al., 2018).

Accordingly, the present study adopted a theory-informed exploratory approach. Rather than testing prespecified directional hypotheses, prior theory and empirical evidence concerning personality, self-regulation, learning behaviors, and emotional responses to digital learning were used to guide the selection of focal constructs and the interpretation of emerging patterns. An explanatory sequential mixed-methods design was employed to: (1) examine associations among personality characteristics, platform-recorded learning behaviors, and learning outcomes following virtual simulation training; (2) explore the cognitive, affective, behavioral, and environmental experiences reported by learners with different personality profiles through the Zaltman Metaphor Elicitation Technique (ZMET); and (3) integrate the quantitative and qualitative findings within Bandura’s triadic reciprocal determinism framework.

By combining quantitative associations with qualitative accounts of learners’ experiences, this study seeks to move beyond simply asking whether personality differences are related to learning outcomes. Instead, it explores how learners with different personality characteristics may engage with the same virtual simulation environment in different ways. The findings may contribute to a more process-oriented understanding of virtual simulation learning and provide preliminary evidence for instructional support that is responsive to variation in learners’ self-regulation, emotional experiences, and patterns of engagement, without treating personality as a fixed determinant of performance.

## 2 Methods

### 2.1 Study design

This study employed an explanatory sequential mixed-methods design, with Bandura’s triadic reciprocal determinism serving as the overarching theoretical framework. The study was conducted in two sequential phases, with the quantitative findings informing participant selection and the focus of the subsequent qualitative inquiry.

In the quantitative phase, a one-group pre-test–post-test design was used to examine observed changes in fall-management knowledge following virtual simulation training and to explore associations among personality characteristics, platform-recorded learning behaviors, and learning outcomes. The quantitative phase was exploratory and was intended to identify patterns of association rather than establish causal effects.

Following the quantitative phase, participants were grouped according to their dominant personality tendency. Within each personality group, participants with relatively high knowledge gains were purposively selected as information-rich cases for the qualitative phase. In-depth interviews using the Zaltman Metaphor Elicitation Technique (ZMET) were then conducted to explore how high-gain learners with different personality tendencies experienced the cognitive, affective, behavioral, and environmental processes of virtual simulation learning.

The two phases were integrated by connecting the quantitative findings to qualitative participant selection and by merging the quantitative and qualitative results during interpretation. Bandura’s triadic reciprocal determinism provided the overarching framework for interpreting the relationships among personal characteristics, learning behaviors, and environmental experiences across the two phases.

### 2.2 Participants

#### 2.2.1 Quantitative sample

Sample size was calculated using G*Power 3.1 software. Referring to relevant literature (Horii et al., 2021), a two-tailed paired-sample t-test was assumed with *α* = 0.05 and (1-β) = 0.95. Aiming to detect a small-to-medium effect size (dz = 0.35), the required sample size was estimated to be 109 participants. Accounting for a potential 20% attrition rate, the final target sample was determined to be 137 standardized training nurses.

Inclusion criteria were: (a) currently undergoing a mandatory, hospital-based standardized training program lasting at least 24 months; and (b) providing informed consent and voluntarily participating in the study. Exclusion criteria were: inability to participate in the standardized training program for ≥14 days during the study period due to job transfer, sick leave, or personal leave.

#### 2.2.2 Qualitative sample

Following completion of the quantitative phase, quantitatively informed stratified purposive sampling was used to select participants for the qualitative phase. All participants were first grouped according to their dominant personality tendency. Within each personality group, participants were then ranked according to their knowledge gain scores, and those with relatively high gains were purposively selected as information-rich cases.

An initial qualitative sample of approximately 15 participants was considered appropriate based on the research team’s previous qualitative research experience. The final sample comprised 17 participants: three from each of the Conscientiousness, Agreeableness, Extraversion, and Open-Mindedness groups, and five from the Neuroticism group. The adequacy of the final sample was assessed according to data saturation, defined as the point at which no substantially new constructs emerged from the interview data.

The qualitative phase focused on exploring how high-gain learners with different personality tendencies experienced the cognitive, affective, behavioral, and environmental processes of virtual simulation learning.

Inclusion criteria were: (a) completion of the quantitative phase and the virtual simulation training; (b) selection as a relatively high-gain case within the participant’s personality group; and (c) provision of informed consent for the qualitative interview. Participants who were unable to articulate their experiences and feelings during the image-elicitation interview were excluded.

### 2.3 Data collection

#### 2.3.1 Quantitative phase

##### 2.3.1.1 Demographic questionnaire

A self-designed demographic questionnaire was developed by the researchers based on a literature review. The questionnaire collected information on the following variables: gender, age, hometown origin (urban/rural), educational background, average academic scores during nursing school, previous online learning experience, average daily online learning time, and preferred learning medium.

##### 2.3.1.2 Big Five Inventory-2 (BFI-2) Chinese version

Personality traits were assessed using the Chinese version of the Big Five Inventory-2 (BFI-2), which was translated and validated by Zhang et al. (2021). The instrument measures five broad personality domains: Extraversion, Agreeableness, Conscientiousness, Neuroticism, and Open-Mindedness. Each domain comprises 12 items, resulting in a total of 60 items, with 30 positively worded and 30 negatively worded items. Items are rated on a 5-point Likert scale ranging from 1 (strongly disagree) to 5 (strongly agree). In the present study, the questionnaire demonstrated acceptable internal consistency. The *α* coefficients for the five domains were as follows: Extraversio*n* = 0.795, Agreeableness = 0.826, Conscientiousness = 0.891, Neuroticism = 0.860, and Open-Mindedness = 0.764.

##### 2.3.1.3 Knowledge assessment test on fall management for nurses

The test content was designed based on the core knowledge points and operational standards covered in the virtual simulation experiment titled “Assessment and Emergency Management of Falls in Older Adults,” and was grounded in authoritative textbooks and monographs in the field of geriatric nursing (Gao et al., 2024; Xu and Zhou, 2024). After initial development, the test was reviewed by two clinical nursing experts and one nursing education specialist. The test items were revised after the first round and resubmitted to the same expert panel for a second review. After two rounds of expert review and item revision, all items achieved an item-level content validity index (I-CVI) of 1.00, indicating complete agreement among the three experts regarding the content relevance of the final items. Subsequently, the 50-item test was pilot-tested with 16 standardized training nurses. The mean item difficulty index was 0.624 (range, 0.000–1.000), and the preliminary KR-20 coefficient was 0.783. Three items were answered correctly by all participants, whereas one item was answered incorrectly by all participants. The test assessed three core competency dimensions: (a) emergency assessment and interventions; (b) medication decision-making and safety; and (c) clinical judgment and subsequent management. Based on feedback from participants in a pilot study, who expressed a preference for reducing academic burden, the test primarily consisted of single-answer and multiple-answer multiple-choice questions. The test comprised 50 items in total: 43 single-answer multiple-choice questions, with 2 points awarded for each correct answer and 0 points for each incorrect answer, and 7 multiple-answer multiple-choice questions, for which full points were awarded only when all correct options were selected, while partial or incorrect selections received 0 points. The maximum possible score was 100 points, and the passing score was set at 60 points. Higher total scores indicated more robust knowledge of fall management among standardized training nurses. This test was administered to standardized training nurses as both the pre-test and post-test before and after completing the National Virtual Simulation Experimental Teaching Platform course.

##### 2.3.1.4 Platform-recorded learning indicators

The National Virtual Simulation Experimental Teaching Platform automatically recorded participants’ learning activities and performance. The indicators extracted for analysis included the total number of learning sessions, average simulation score, and the timing of initial engagement with the training program.

##### 2.3.1.5 Pre-test and preparation

The researchers coordinated with the nursing administration department during the study period with the goal of minimizing the risk that training activities with substantial content overlap with geriatric fall management would be scheduled, thereby attempting to reduce potential confounding. The demographic questionnaire was distributed via the Wenjuanxing platform, a widely used online survey tool in China. All standardized training nurses enrolled in the study completed a uniform pre-test administered in a face-to-face setting to assess their baseline knowledge of fall prevention and emergency management, thereby establishing a knowledge baseline prior to the training program. Subsequently, all participants received standardized orientation regarding the purpose and significance of the study, as well as instructions for using the virtual simulation platform.

##### 2.3.1.6 The virtual simulation training program

The training program consisted of the virtual simulation experiment titled “Assessment and Emergency Management of Falls in Older Adults,” developed by Central South University and hosted on the National Virtual Simulation Experimental Teaching Platform. The simulation was structured around a clinical case of an older adult patient experiencing a fall, encompassing five typical post-fall scenarios: Unrestricted mobility; Conscious or unconscious state; Absence of spontaneous breathing; Cervical-thoracic-lumbar spine injury; Limb trauma. The program was organized into sequential modules corresponding to initial assessment, further assessment, and emergency management. Participants were required to accurately assess the patient’s condition within each virtual scenario and perform appropriate emergency procedures. Core tasks included both theoretical knowledge and practical skills, such as assisting the patient to stand independently, performing hands-only cardiopulmonary resuscitation (CPR), controlling bleeding, applying bandages, immobilizing injuries, and safely transferring the patient.

A task-oriented learning model was adopted, allowing participants to determine their own learning pace, including the number of learning sessions and the duration of each session, based on their individual needs. All learning activities and performance data were automatically recorded by the National Virtual Simulation Experimental Teaching Platform.

##### 2.3.1.7 Post-test and data collection

One week after completion of the one-month virtual simulation training program, all participants completed a face-to-face post-test using the same fall-management knowledge assessment administered at baseline. The interval was intended to reduce the influence of immediate recall and assess short-term knowledge retention. During the same session, participants completed the Chinese version of the Big Five Inventory-2 (BFI-2); therefore, personality traits were assessed after the training program.

#### 2.3.2 Qualitative phase

The qualitative phase employed the Zaltman Metaphor Elicitation Technique (ZMET). Based on findings from a pilot study, and to address potential difficulties participants might experience in identifying images that adequately represented their learning experiences, a standardized image library comprising 60 images was developed following a review of the relevant literature (Li, 2023; Zhao, 2024). Individual interviews lasted approximately 50–70 min and were audio-recorded with participants’ permission. During the interviews, the laddering technique was used to probe the deeper meanings underlying participants’ image selections and descriptions. Data collection continued until no substantially new constructs emerged from subsequent interviews. All interviewers had prior qualitative research experience and had received specialized training in qualitative methods.

### 2.4 Data analysis

#### 2.4.1 Quantitative data

Statistical analysis was performed using SPSS version 26.0. Demographic characteristics were described using frequencies and constituent ratios. Histogram analysis indicated that the core variables—pre-test scores, post-test scores, and knowledge gain scores —were normally distributed. The numbers of standardized training nurses with each personality trait were described using frequencies and percentages. Pre-test and post-test scores, as well as learning behavior data (number of learning sessions and learning duration), were described using means and standard deviations. The five BFI-II domains were analyzed as continuous variables, whereas dominant personality grouping was used only as an exploratory simplification for secondary comparisons and qualitative sampling, not as a definitive typology.

Pearson correlation analyses were conducted as exploratory descriptive analyses and were not used as criteria for variable selection. Hierarchical multiple linear regression was performed with post-test knowledge score as the outcome. Pre-test knowledge score and relevant covariates were entered in the first block, the five continuous BFI-II domain scores in the second block, and learning sessions and average simulation score in the third block. Variables were entered using the Enter method based on theoretical relevance rather than bivariate statistical significance.

#### 2.4.2 Qualitative data

Within 24 h after each interview, the audio recordings were transcribed verbatim by one researcher. The transcripts were then reviewed by a second researcher to verify accuracy and ensure consistency with the audio recordings. Qualitative coding and construct extraction were conducted independently by two researchers using a primarily inductive approach without an *a priori* formal codebook. Disagreements were resolved through discussion with a third researcher until consensus was reached. Bandura’s triadic reciprocal determinism was applied during mixed-methods integration and interpretation rather than as an a priori coding framework.

##### 2.4.2.1 Construct extraction and standardization

Each interview transcript was carefully read in depth. Key words and phrases expressing participants’ core feelings and thoughts were extracted from: (a) their “story descriptions” of the images they brought, (b) their descriptions of imagined missing images, (c) their sensory impressions. Expressions with identical or similar meanings were consolidated into standardized “constructs.”

##### 2.4.2.2 Construction of individual mental maps

Based on the interview texts, the logical relationships among constructs were identified. Using these constructs as nodes and the logical relationships as connections, an “individual mental map” was drawn for each participating standardized training nurse to visually represent their unique personal learning cognitive network. Each preliminary mental map was presented to the corresponding participant for confirmation to ensure it accurately reflected their inner perceptions, thereby safeguarding the validity of the analysis at the individual level.

##### 2.4.2.3 Construction of group consensus map

The individual mental maps of all participating nurses were integrated. Following ZMET’s convergence principle (Zaltman, 1997), only constructs and relationships that were mentioned by at least one-third of the interviewees were retained. A “group consensus map” was then drawn to reflect the shared experiences of the participant group. Through thematic extraction and integration, the core experiential themes shared by all participants were identified from the group consensus map.

### 2.5 Quality control

To maximize control over confounding factors and ensure measurement validity in the quantitative phase, the following measures were implemented: answers were not provided for either the pre-test or post-test; each test was configured as a single access with items presented in random order; all questionnaire items were set as mandatory; and submissions from the same IP address were restricted to once only, thereby ensuring data completeness and uniqueness.

Additionally, this study did not establish any *a priori* theoretical hypotheses during the research process. The researchers maintained an open and neutral stance throughout, striving to minimize the influence of personal subjective tendencies on the research process.

To enhance the trustworthiness of the qualitative phase, the following specific measures were adopted based on Lincoln and Guba (1985) criteria:

Credibility: Consensus and discrepancies in the data were systematically identified and confirmed through: (a) cross-checking interview audio recordings, (b) independent coding by two researchers, and (c) participant feedback on preliminary analyses.
Dependability: Reflective notes were written immediately after each interview, documenting contextual information such as participants’ nonverbal behaviors, attitudes toward participation, and the interview environment, thereby providing comprehensive background information for data analysis.
Transferability: When presenting the research findings, rich contextual descriptions and verbatim quotations from participants were provided, elaborating on the connotations of each theme in detail. This enhances the reference value of the study’s conclusions for other similar contexts.
Confirmability: The researchers maintained ongoing reflective diaries throughout the research process, documenting their thought trajectories, potential presuppositions, and emotional responses. Through this reflective practice, personal biases were identified and their potential influence on the research findings was minimized.

### 2.6 Data integration

Through triangulation, the findings from the quantitative and qualitative phases were compared, interpreted, and integrated. In accordance with the explanatory sequential mixed-methods design, the qualitative phase was conducted to further explain and enrich the quantitative findings. The integration process focused on identifying areas of convergence, complementarity, and expansion between the two datasets. To enhance the transparency of this integration, the quantitative results, qualitative explanations, and their corresponding interpretations within Bandura’s Triadic Reciprocal Determinism framework were systematically summarized in Table 1. All authors participated in discussions throughout the integration process to ensure the consistency and credibility of the final interpretations.

**Table 1:** Joint display of quantitative and qualitative findings within the framework of triadic reciprocal determinism.

Quantitative findings	Qualitative explanations	Corresponding triadic reciprocal determinism framework	Integrated interpretations
Conscientiousness: In the secondary exploratory comparison, the Conscientiousness group showed a larger mean knowledge gain than the Neuroticism group. When the five personality dimensions were analyzed as continuous variables, Conscientiousness was positively associated with post-test knowledge in Model 2, but this association was no longer statistically significant after learning sessions and average simulation scores were added in Model 3.	Participants with a dominant Conscientiousness tendency frequently described self-discipline, planning, systematic review, repeated practice, and systematic organization of knowledge.	Person-behavior association	The quantitative and qualitative findings were consistent with an association between Conscientiousness and self-regulated learning behaviors, including planning and repeated practice. However, the attenuation of the Conscientiousness coefficient in the final regression model does not establish that these behaviors mediated an effect of personality on knowledge outcomes.
Neuroticism: In the secondary exploratory comparison, the Neuroticism group showed a smaller mean knowledge gain than the Conscientiousness group. Neuroticism was not independently associated with post-test knowledge in the hierarchical regression models.	Participants with a dominant Neuroticism tendency frequently described anxiety, confusion, time pressure, additional burden, fear of evaluation, and perfectionistic concerns.	Person-environment interplay associated with emotional and behavioral responses	The exploratory group comparison showed a smaller mean knowledge gain among participants classified as having a dominant Neuroticism tendency. Qualitative accounts highlighted anxiety, confusion, time pressure, and evaluation concerns, but the study did not establish that these experiences caused reduced engagement or lower knowledge scores.
Learning sessions: The number of learning sessions was positively correlated with knowledge gain. It also remained positively associated with post-test knowledge in the final regression model after baseline knowledge, covariates, personality dimensions, and average simulation scores were considered.	Participants distinguished between “meaningful repetition” and “mechanical learning.” Meaningful repetition involved reflection, knowledge internalization, and progressive consolidation, whereas mechanical learning involved repeated task completion with limited processing of the underlying clinical rationale.	Behavior-outcome association	A greater number of learning sessions was associated with higher knowledge outcomes. The qualitative findings further suggested that the quality of repetition may also be relevant; increasing session frequency alone should not be interpreted as sufficient to improve learning outcomes.
Average simulation scores: Average simulation scores were positively correlated with knowledge gain and remained positively associated with post-test knowledge in the final regression model.	Participants described clear instructions, timely feedback, systematic knowledge organization, task-oriented scenarios, and immersion as features that helped them understand and adjust their learning processes.	Interplay between the learning environment, behavioral engagement, and cognitive processing	Higher simulation scores were associated with higher post-test knowledge. Participants’ accounts suggested that structured scenarios, feedback, and immersion were experienced as supportive features, although these findings do not establish a causal mechanism linking platform characteristics, simulation performance, and knowledge improvement.
Agreeableness: The Agreeableness group showed the second-highest mean knowledge gain descriptively, but no statistically significant pairwise differences involving this group were identified. Agreeableness was not independently associated with post-test knowledge in the hierarchical regression.	Participants with a dominant Agreeableness tendency frequently described comforting anxious peers, sharing learning strategies, studying together, providing assistance, and contributing to a collaborative atmosphere.	Person-behavior-environment interplay	Agreeableness was not associated with individual knowledge outcomes, but the qualitative accounts suggested that it may be related to supportive peer behaviors and a collaborative learning climate. Because peer-network effects and group-level learning outcomes were not directly measured, this interpretation remains preliminary.
Overall pre-test-post-test comparison: Post-training knowledge scores were significantly higher than pre-training scores following the virtual simulation program.	Participants described the platform as a lower-risk practice environment that allowed them to attempt clinical decisions repeatedly, receive feedback, correct mistakes, and apply knowledge without the possibility of harming actual patients.	Environment-behavior-outcome association	Knowledge scores were higher after training than before training. Participants’ experiences were compatible with a supportive role of the virtual simulation environment, but the observed improvement cannot be attributed exclusively to the program because the study did not include randomization or a control group.

Dominant personality grouping was used as an exploratory analytical simplification and for qualitative sampling rather than as a definitive personality typology. The linkages presented in this joint display represent theory-informed interpretations derived from mixed-methods integration. They should not be interpreted as empirically tested causal, directional, mediation, or reciprocal pathways.

### 2.7 Ethical considerations

This study protocol adhered to the principles of the Declaration of Helsinki and received ethical approval from the relevant institutional review board. The study was also registered in a national research registry.

## 3 Results

### 3.1 Quantitative findings

#### 3.1.1 Demographic characteristics and personality type distribution of participants

A total of 140 standardized training nurses participated in this study. After excluding two participants due to quality control concerns (identical responses to all questions) and 10 participants who withdrew from the online virtual simulation learning platform during the study, 128 valid responses were included in the final analysis, yielding an effective response rate of 91.43%, which met the required sample size.

Among the 128 standardized training nurses, 88.3% (*n* = 113) were female and 11.7% (*n* = 15) were male. Regarding hometown origin, 64.8% (*n* = 83) were from rural areas and 35.2% (*n* = 45) from urban areas. In terms of educational background, 81.3% (*n* = 104) held an associate degree and 18.8% (*n* = 24) held a bachelor’s degree.

The distribution of average academic scores during nursing school was as follows: >90 points, 12.5% (*n* = 16); 80–90 points, 45.3% (*n* = 58); 70–79 points, 34.4% (*n* = 44); 60–69 points, 7.0% (*n* = 9); and <60 points, 0.8% (*n* = 1).

A total of 69.5% (*n* = 89) of participants had prior experience with online learning. Among these 89 participants, the distribution of average daily online learning time was: <2 h, 55.1% (*n* = 49); 2–4 h, 36.0% (*n* = 32); 4–6 h, 6.7% (*n* = 6); and >6 h, 2.2% (*n* = 2). Their preferred learning media were: recorded courses, 63.6% (*n* = 56); live-streamed courses, 15.9% (*n* = 14); text-and-graphic self-study, 15.9% (*n* = 14); and virtual simulation platforms, 4.5% (*n* = 4).

Regarding the distribution of personality types, Conscientiousness accounted for 29.7% (*n* = 38), followed by Neuroticism (28.1%, *n* = 36), Extraversion (23.4%, *n* = 30), Agreeableness (13.3%, *n* = 17), and Open-Mindedness (5.5%, *n* = 7).

#### 3.1.2 Descriptive statistics of key variables

The results showed that standardized training nurses with Conscientiousness and Agreeableness personality tendencies achieved the highest knowledge gain scores (9.34 ± 7.30 and 9.29 ± 5.58, respectively), while those with Neuroticism exhibited the lowest knowledge gain scores (4.64 ± 6.80). Pre-test scores were comparable across the five personality types; however, post-test scores indicated that nurses with Neuroticism showed relatively smaller improvements (Table 2).

**Table 2:** Descriptive statistics of standardized training nurses’ scores by personality type (*N* = 128).

Personality type	*n*	Pre-test score	Post-test score	Knowledge gain score
Conscientiousness	38	70.74 ± 7.23	80.39 ± 8.84	9.65 ± 7.30
Agreeableness	17	69.65 ± 7.72	78.94 ± 7.60	9.29 ± 5.55
Open-mindedness	7	68.29 ± 10.16	77.71 ± 7.78	9.00 ± 9.33
Extraversion	30	72.37 ± 6.32	79.73 ± 6.05	7.36 ± 7.23
Neuroticism	36	71.06 ± 7.16	75.19 ± 5.10	4.13 ± 6.80
Total	128	70.93 ± 7.21	78.44 ± 7.28	7.51 ± 7.22

#### 3.1.3 Observed pre–post changes in knowledge scores of online learning among standardized training nurses

Paired-sample t-test results showed that after completing the online virtual simulation training program, standardized training nurses’ post-test scores (78.44 ± 7.28) were significantly higher than their pre-test scores (70.93 ± 7.21), with a mean improvement of 7.51 points (*t* = −11.32, *p* < 0.001, 95% CI: −8.82 to −6.20). Detailed results are presented in Table 3.

**Table 3:** Paired-sample *t*-test results of pre-test and post-test knowledge scores among Standardized training nurses (*N* = 128).

Paired comparison	Mean difference	SD	t	df	*P*	95% CI
Pre-test – Post-test	−7.51	7.50	−11.32	127	<0.001	−8.82 ~ −6.20

#### 3.1.4 Exploratory comparison across dominant personality tendencies

As a secondary exploratory analysis, a one-way ANOVA indicated a statistically significant difference in knowledge gains among the five dominant personality groups, *F*(4, 123) = 2.49, *p* = 0.046, η^2^ = 0.075. Tukey’s HSD *post hoc* test showed that the Conscientiousness group had greater knowledge gains than the Neuroticism group [mean difference = 4.70, 95% CI (0.16, 9.25), adjusted *p* = 0.039]. No other pairwise differences were statistically significant. Detailed results are presented in Table 4.

**Table 4:** One-way ANOVA results of knowledge gain scores by dominant personality tendency among standardized training nurses.

Source of variation	Sum of squares	df	Mean square	F	*P*
Between groups	496.86	4	124.22	2.49	0.046*
Within groups	6125.35	123	49.80		
Total	6622.22	127			

*P < 0.05.

#### 3.1.5 Correlation analysis between knowledge gain scores and related variables

Correlation analysis revealed that knowledge gain scores were significantly positively correlated with both the number of learning sessions and average simulation scores (both *p* < 0.01).

In addition, the start time of learning (i.e., later start) was significantly negatively correlated with the number of learning sessions (*p* < 0.05) (Table 5).

**Table 5:** Correlation analysis between knowledge gain scores and related variables among standardized training nurses (*N* = 128).

Variable	Knowledge gain scores	Number of learning sessions	Average simulation scores	Start time of learning
Knowledge gain scores	1			
Number of learning sessions	0.51**	1		
Average simulation scores	0.43**	0.43**	1	
Start time of learning	0.05	−0.21*	−0.09	1

Start time of learning was coded as: 1 = started before the first week of the study period; 2 = started in Week 2 or 3; 3 = started after Week 3. *P < 0.05; **P < 0.01 (two-tailed).

#### 3.1.6 Hierarchical multiple linear regression analysis

Hierarchical regression showed that baseline knowledge and covariates accounted for 29.3% of the variance in post-test knowledge scores. Adding the five continuous personality dimensions accounted for an additional 8.3% of the variance (∆R^2^ = 0.08, *p* = 0.011), with Conscientiousness positively associated with post-test knowledge at this stage (*β* = 0.25, *p* = 0.035).

Learning behaviors accounted for a further 19.6% of the variance (∆R^2^ = 0.20, *p* < 0.001). The final model explained 57.3% of the variance in post-test knowledge scores (adjusted R^2^ = 0.53). Pre-test score (*B* = 0.53, *p* < 0.001), number of learning sessions (*β* = 0.30, *p* < 0.001), and average simulation score (*β* = 0.25, *p* = 0.001) remained statistically significant, whereas none of the five personality dimensions remained significant in the final model (see Table 6).

**Table 6:** Hierarchical regression analysis for learning outcomes (*N* = 128).

Variables	Model 1			Model 2			Model 3		
	B	SE	β	B	SE	β	B	SE	β
Pre-test knowledge	0.45	0.08	0.46**	0.48	0.08	0.48**	0.53	0.07	0.53**
Gender	2.54	1.72	0.11	3.02	1.70	0.14	2.26	1.43	0.10
Educational background	1.57	1.49	0.09	1.08	1.45	0.06	0.32	1.21	0.02
Average academic scores during nursing school	0.80	0.68	0.09	0.37	0.67	0.04	0.14	0.56	0.02
Previous online learning experience	−2.48	1.21	−0.16*	−1.75	1.22	−0.11	−2.03	1.03	−0.13
Extraversion	—	—	—	0.19	0.11	0.17	0.09	0.09	0.08
Agreeableness	—	—	—	−0.09	0.12	−0.08	0.00	0.10	0.00
Conscientiousness	—	—	—	0.24	0.11	0.25*	0.18	0.1	0.18
Neuroticism	—	—	—	−0.05	0.11	−0.06	−0.02	0.09	−0.02
Open-mindedness	—	—	—	−0.11	0.12	−0.09	−0.07	0.10	−0.06
Number of learning sessions	—	—	—	—	—	—	0.48	0.11	0.30**
Average simulation scores	—	—	—	—	—	—	0.30	0.08	0.25**
R^2^	0.29			0.38			0.57		
Adjusted R^2^	0.26			0.32			0.53		
F	10.12**			7.07**			12.84**		

*P < 0.05; **P < 0.01.

### 3.2 Qualitative findings

A total of 17 standardized training nurses participated in the interviews. The majority were female (82.35%), and ages ranged from 20 to 22 years. Detailed characteristics of the participants are presented in Table 7.

**Table 7:** Demographic and background characteristics of interview participants.

ID	Personality type	Gender	Pre-test score	Post-test score	Number of learning sessions
01	Conscientiousness	Female	64	90	28
02	Conscientiousness	Male	70	90	14
03	Conscientiousness	Female	58	88	6
04	Agreeableness	Female	76	92	8
05	Agreeableness	Female	80	92	4
06	Agreeableness	Female	83	88	33
07	Open-Mindedness	Female	60	78	6
08	Open-Mindedness	Female	64	80	16
09	Open-Mindedness	Female	80	88	4
10	Extraversion	Female	74	90	10
11	Extraversion	Female	62	75	10
12	Extraversion	Female	64	84	7
13	Neuroticism	Female	64	72	3
14	Neuroticism	Female	60	76	5
15	Neuroticism	Male	66	74	5
16	Neuroticism	Female	72	80	4
17	Neuroticism	Female	66	74	4

Number of learning sessions refers to the total times each participant completed the virtual simulation scenarios.

Based on the analysis of metaphor elicitation technique (ZMET) interview data from 17 standardized training nurses, this study constructed a consensus map of learning experiences in the online virtual simulation experimental teaching platform. A total of 17 raw constructs and 15 relational constructs were extracted, which were further synthesized into five final constructs.

#### 3.2.1 Raw constructs: representations of initial actions and direct feelings

The detailed content is presented in Table 8.

**Table 8:** Raw constructs and their meanings among standardized training nurses of different personality types in the online virtual simulation platform.

Personality type	Raw construct	Interpretation	Verbatim quote
Agreeableness	Mutual support	Learners support each other, fostering a collaborative learning atmosphere that emphasizes collectivism and teamwork.	ID.05: “At that time, another friend was also studying on this platform. She felt very anxious about the virtual simulation learning, so I actively comforted her and told her it wasn’t that difficult—we could learn together.”
	External help	Reliance on external resources or guidance from others, such as the research team recommending the platform or others explaining procedures.	ID.04: “The research team assigning this little learning task was like bait, luring me out.”
	Trust	Belief that the platform recommended by the research team is reliable and valuable; willingness to follow arrangements.	ID.04: “I trusted the platform and followed the instructions step by step, without worrying about making mistakes.”
Extraversion	Clear instructions	Perception of the clarity of instructions from the platform or task assigners; clear instructions facilitate smooth learning.	ID.11: “I learned the relevant content under the active guidance of the platform.”
	Free teaming	Preference for autonomously choosing learning partners, emphasizing autonomy and flexibility; preference for learning with familiar peers.	ID.12: “I completed the virtual simulation learning tasks with my friends. I think learning with friends creates a positive and uplifting atmosphere, and in that state, my learning efficiency is higher.”
	Authoritative resources	Belief that the knowledge provided by the platform is authoritative and more trustworthy than other online resources.	ID.10: “Previously, I mostly acquired knowledge from various online platforms, where I had to identify and verify information myself. But I believe the knowledge on this platform is produced by professional academic institutions, so I consider it relatively authoritative.”
Conscientiousness	Self-discipline	Emphasis on self-management; proactively sets learning plans and strictly adheres to them.	ID.01: “When learning on the virtual simulation platform, I set alarms to remind myself to study. When the alarm went off, I knew it was time to go to the platform and start learning.”
	Planning	Emphasizes organization in the learning process; tends to systematically arrange tasks and incorporate learning into daily schedules.	ID.03: “After completing the virtual simulation learning, I added offline learning tasks related to the knowledge I had not fully understood to my study plan.”
	Systematic knowledge	Recognition of the platform’s well-organized and systematic content; clear modules facilitate knowledge construction.	ID.02: “In the virtual simulation, patients presented different conditions after falling, and the platform had corresponding modules for different conditions.” I think this structured design is very user-friendly and helpful for building my knowledge framework.”
	Repetition	Consolidates knowledge through repeated practice; demonstrates high standards for knowledge mastery.	ID.01: “To prevent knowledge from fading, I reviewed it whenever I had free time. Sometimes I studied it several times a day.”
Open-Mindedness	Exploration	Curiosity about new knowledge and fields; actively seeks to explore unfamiliar content.	ID.09: “I was curious about this new way of learning—virtual simulation. It was this desire to explore that made me start learning immediately when the task was assigned.”
	Immersion	Deep engagement in the learning process; the virtual format creates a sense of presence, enabling an enjoyable deep learning experience.	ID.09: “This learning method pulled me directly into the constructed scenario. At that moment, I was the nurse inside it, performing the relevant operations. It gave me a strong sense of presence and immersion.”
	Sense of achievement	Gains satisfaction and a sense of achievement from learning, such as successfully constructing a knowledge system, saving a patient in the simulation, or mastering the content.	ID.07: “Even though it was just a virtual simulation experiment, when I, as a nurse in the platform, helped the patient receive treatment through my actions, I also felt a sense of pride.”
Neuroticism	Extra burden	Perceives the learning task as exceeding personal capacity; views it as an additional burden on top of existing work and study pressures.	ID.13: “I’m already busy with work; how could I possibly have time for extra studying? I felt I had no time for the virtual simulation experiment—my time was being squeezed.”
	Time pressure	Anxiety caused by time constraints, such as approaching deadlines, conflicts with other commitments, or difficulty scheduling learning.	ID.16: “As the deadline approached, I saw classmates posting pictures of their learning. There were only 10 days left, and I had not even started. I felt extremely depressed inside.”
	Confusion	Feeling puzzled by the learning content or knowledge structure; lacks organization and direction, like tangled thoughts.	ID.17: “Not understanding the knowledge felt like a bunch of tangled earphone wires—it looked completely messy and disorganized. I simply could not sort out my thoughts.”
	Anxiety	Exhibits, and worry when facing learning tasks, such as concern about scores, fear of others’ judgments, or perceived lack of knowledge.	ID.15: “I did not know what the experimental scores represented. I was afraid that after submitting my learning, I would end up with a poor score, and that would make others look at me differently.”

#### 3.2.2 Relational constructs

The relational constructs were derived from the interactions of raw constructs, reflecting more comprehensive psychological states, behavioral patterns, and environmental characteristics during standardized training nurses’ learning processes. A total of 15 relational constructs were identified in this study, which were categorized into four types based on their functional orientation, as illustrated in Table 9.

**Table 9:** Classification and functional orientation of relational constructs.

Category	Relational constructs	Functional orientation
Learning environment characteristics	Mutual support, External help, Learning atmosphere, Timely feedback	Refers to the learning environment in which standardized training nurses are situated, including peer collaboration, introduction of external resources, overall atmosphere, and the platform’s instructional feedback mechanisms.
Knowledge processing	Clear organization, knowledge credibility, knowledge internalization, learning habits, Mechanical learning	Refers to standardized training nurses’ processing of knowledge, including judgments about knowledge structure, assessments of knowledge reliability, transformation of knowledge from external to internal, and the patterning and solidification of learning behaviors.
Learning drivers	Exploration desire, professional identity	Refers to the psychological forces driving standardized training nurses’ learning, including curiosity and impulse to seek new knowledge, as well as the pursuit of value in continuously affirming their professional identity through specialized learning.
Learning stressors	Learning frustration, Learning burden, employment pressure, perfectionism	Refers to various difficulties and pressures encountered by standardized training nurses during the learning process, including task obstruction, task consumption, concerns about future career development, and excessively high self-expectations.

#### 3.2.3 Final constructs

The final constructs represent the highest level of abstraction derived from all raw constructs and relational constructs, constituting the core dimensions of standardized training nurses’ online virtual simulation learning experiences. Due to their rich connotations, some raw constructs were simultaneously attributed to multiple final constructs and are presented below according to their primary dimensional associations (Table 10).

**Table 10:** Final constructs and their constituent constructs.

Final construct	Constituent constructs
Learning support	Interaction, help, trust, free teaming, clear instructions, Authoritative academic resources, mutual support, external help, Timely feedback, learning atmosphere
Knowledge construction	Systematic platform knowledge, repetition, exploration, clear organization, knowledge credibility, knowledge internalization, learning habits
Emotional experience	Immersion, sense of achievement, anxiety, confusion, learning frustration, perfectionism
Learning motivation	Self-discipline, planning, exploration, exploration desire, Professional identity
External learning pressure	Time pressure, extra burden, employment pressure, learning burden

#### 3.2.4 Consensus map of learning experiences in the virtual simulation platform

The three levels of constructs described above form a hierarchical structure ranging from concrete to abstract. The raw constructs are situated at the top level, serving as the foundational materials of the consensus map. The relational constructs occupy the middle level, derived from the interactions of raw constructs. The final constructs reside at the bottom level, representing a high-level abstraction of the preceding two levels. Constructs connected by lines in the map indicate direct associative relationships, collectively constituting the overall picture of standardized training nurses’ online virtual simulation learning experiences. The consensus map is presented in Supplementary file 1.

## 4 Discussion

### 4.1 Overview of findings within the triadic reciprocal framework

Taken together, the findings are most appropriately interpreted as theory-informed patterns of association rather than as validated causal pathways. Bandura’s triadic reciprocal determinism provides an overarching framework for organizing the relationships among personal characteristics, learning behaviors, and the virtual simulation environment. Within this framework, person, behavior, and environment are viewed as mutually relevant components of learning rather than as isolated determinants.

The quantitative findings showed that platform-recorded learning indicators, particularly the number of learning sessions and average simulation scores, were the strongest proximal correlates of post-test knowledge. By contrast, none of the five continuous personality dimensions remained statistically significant after learning behaviors were entered into the final regression model. The qualitative findings provided complementary insights by showing that different personality tendencies tended to co-occur with different behavioral, emotional, and interpersonal experiences. Conscientiousness was commonly described alongside planning, self-discipline, and repeated practice; Neuroticism was accompanied by anxiety, confusion, time pressure, and fear of evaluation; and Agreeableness was reflected in peer support and collaborative learning behaviors.

These findings suggest that personality characteristics may be relevant to how learners engage with the virtual simulation environment, but they do not establish that personality directly or indirectly causes particular learning outcomes. Learners also appeared to contribute to their learning environment through their own behaviors, particularly through peer support and collaboration. Table 1 summarizes the convergence and complementarity between the quantitative and qualitative findings. The relationships presented in the table should be understood as interpretive linkages derived from mixed-methods integration rather than as empirically tested causal, directional, or mediation pathways.

### 4.2 Observed knowledge improvement following virtual simulation training

Post-training knowledge scores were significantly higher than pre-training scores, indicating an observed improvement in fall-management knowledge following the virtual simulation program. This finding is consistent with previous systematic reviews and empirical studies reporting that virtual simulation is associated with improved clinical knowledge among nursing students and healthcare professionals (Woon et al., 2021; Liu et al., 2023). Recent studies have also demonstrated the feasibility of virtual simulation in fall prevention and other nursing education contexts (Yan et al., 2025; Park et al., 2025).

However, because the present study used a one-group pre-test–post-test design without randomization or a control group, the observed score improvement cannot be attributed exclusively to the virtual simulation program. Other influences, including clinical experience, independent review, peer interaction, and familiarity with the assessment, may also have contributed.

The qualitative findings nevertheless provide useful contextual information about how participants experienced the training environment. Participants described the simulation as a lower-risk setting in which they could repeatedly attempt clinical decisions, receive feedback, correct errors, and apply knowledge without the possibility of harming actual patients. This feature may be particularly relevant to fall management, where assessment and emergency interventions can involve substantial consequences in clinical practice. Although psychological safety was not directly measured, participants’ accounts suggest that the absence of real-patient risk may have reduced some of the anxiety associated with trial-and-error learning.

The sequence of attempting a task, receiving feedback, reconsidering one’s understanding, and applying revised knowledge in subsequent attempts is also broadly compatible with experiential learning. However, Kolb’s experiential learning cycle was used only as background for interpreting the simulation process and was not employed as an analytical framework or as an *a priori* structure for qualitative coding.

### 4.3 Personality traits and learning outcomes: exploratory associations

As a secondary exploratory analysis, the Conscientiousness group showed a larger mean knowledge gain than the Neuroticism group in the one-way ANOVA with Tukey’s HSD *post hoc* comparison. This exploratory difference should be interpreted cautiously because the omnibus significance was borderline, subgroup sizes were unequal, and the dominant-trait grouping represented a simplified analytical classification rather than mutually exclusive personality types.

The hierarchical regression produced a more nuanced pattern. When the five personality dimensions were entered as continuous variables, Conscientiousness was positively associated with post-test knowledge in Model 2. However, this association was no longer statistically significant after the number of learning sessions and average simulation scores were entered into the final model. The attenuation of the Conscientiousness coefficient is consistent with the possibility that learning behaviors may partly account for the observed association. Nevertheless, coefficient attenuation alone does not demonstrate mediation. Shared variance, unmeasured confounding, or other relationships among personality, behavior, and learning outcomes may also explain this pattern.

ANOVA and multiple regression also address different questions. The exploratory ANOVA compared mean knowledge gains among simplified dominant-trait groups, whereas the regression assessed the independent association of each continuous personality dimension with post-test knowledge after accounting for baseline knowledge, demographic and educational covariates, and learning indicators. The two analyses should therefore not be expected to yield identical conclusions.

The qualitative findings help contextualize, but do not causally explain, these statistical patterns. Participants with a dominant Conscientiousness tendency frequently described planning, systematic review, self-discipline, and deliberate repetition. These experiences are consistent with previous evidence linking Conscientiousness with self-regulated learning and sustained academic effort (Poropat, 2009; Zhao et al., 2025; Chen et al., 2025). Participants with a dominant Neuroticism tendency more often described anxiety, uncertainty, confusion, time pressure, and concerns about evaluation. These experiences may make engagement more difficult for some learners, although the present study did not demonstrate that Neuroticism caused reduced practice or lower knowledge scores.

From the perspective of triadic reciprocal determinism, the findings suggest an interplay among personal tendencies, behavioral engagement, emotional experience, and environmental conditions. The framework is useful for interpreting why learners exposed to the same simulation platform may experience and use it differently. It should not, however, be taken as evidence that the specific directional pathways proposed in the qualitative interpretation have been statistically established.

### 4.4 Learning engagement and the quality of repetition

The number of learning sessions and average simulation scores remained significantly associated with post-test knowledge in the final regression model. These findings support the relevance of platform-recorded indicators for understanding engagement in virtual learning environments (Li et al., 2024; Osmanli, 2025). At the same time, the qualitative findings indicate that the quantity of engagement may not fully capture its educational quality.

Participants distinguished between meaningful repetition and mechanical learning. Meaningful repetition involved progressively improving procedural familiarity, reconsidering the principles underlying each action, integrating feedback, and consolidating knowledge across successive attempts. Mechanical learning, in contrast, was described as repeatedly completing platform operations without sufficiently processing the underlying clinical rationale.

This distinction offers a possible interpretation of why practice frequency alone may be an incomplete indicator of learning. A high number of sessions may reflect sustained cognitive engagement, but it may also reflect repeated task completion with limited reflection. The present qualitative findings cannot establish that meaningful repetition caused greater knowledge gain, but they suggest that learning analytics should move beyond simple counts of sessions.

Future studies should collect more detailed process indicators, including time spent on individual tasks, error patterns, operation sequences, use of feedback, help-seeking behaviors, and changes across successive attempts. Such data could help distinguish deliberate practice and knowledge construction from superficial repetition.

### 4.5 Agreeableness and the supportive learning climate

Agreeableness was not significantly associated with post-test knowledge in the hierarchical regression, and no statistically significant pairwise differences involving the Agreeableness group were identified in the exploratory group comparisons. This finding is consistent with previous evidence suggesting that the association between Agreeableness and individual academic performance is generally smaller and more context-dependent than the association observed for Conscientiousness (Poropat, 2009; Chen et al., 2022; Mammadov, 2021; Tan et al., 2024).

The qualitative findings nevertheless indicated that Agreeableness may be relevant to the social context of learning. Participants with a dominant Agreeableness tendency frequently described comforting anxious peers, sharing learning strategies, studying together, and fostering a cooperative atmosphere. One participant represented this experience through the metaphor of “a large cactus watering a small cactus,” illustrating the provision of emotional and practical support to another learner.

These accounts suggest a possible association between Agreeableness and supportive peer behaviors. Such behaviors may contribute to a collaborative learning climate, even when Agreeableness is not associated with an individual learner’s knowledge score. This interpretation is compatible with triadic reciprocal determinism because learners may participate in shaping the interpersonal environment in which learning occurs.

However, the present study did not measure peer networks, team-level learning outcomes, knowledge-sharing frequency, or the effects of one participant’s behavior on other learners’ performance. It therefore cannot establish that Agreeableness indirectly improved group learning outcomes or that peer support mediated an association between personality and knowledge gain. The findings should instead be viewed as preliminary qualitative evidence that Agreeableness may be relevant to the social and environmental dimensions of virtual simulation learning.

This interpretation represents a potentially distinctive contribution of the study. It shifts attention from personality as an individual predictor of test performance toward personality-related interpersonal behaviors that may be important in collaborative educational settings. Future studies could examine this possibility using social-network analysis, multilevel modelling, direct measures of peer support, and group-level learning outcomes.

### 4.6 Contextual experiences emerging from person–behavior–environment interplay

The qualitative phase identified two additional experiences that were not readily captured by the quantitative variables: maladaptive perfectionism and employment pressure. These findings should be treated as contextual and hypothesis-generating rather than as established mechanisms.

First, one participant with a dominant Neuroticism tendency described feeling “like a butterfly trapped in a glass bottle” when unable to achieve a perfect simulation score. This account reflected persistent self-criticism, concern about performance, and eventual emotional exhaustion. Previous research has linked perfectionistic concerns with academic stress and test anxiety (Piredda et al., 2025). In the present context, the immediate visibility of simulation scores may have made performance evaluation particularly salient to learners who were already concerned about mistakes. This interpretation is plausible but was not directly tested.

Second, employment pressure emerged as part of the external learning-pressure construct. Participants described concerns that their learning performance might affect future employment opportunities. This experience reflects the dual position of standardized training nurses as both learners and prospective job applicants. Broader employment conditions may therefore form part of the context in which simulation tasks are interpreted, potentially functioning as either motivation or psychological pressure.

Within the triadic reciprocal framework, these accounts illustrate how personal concerns, behavioral responses, and environmental conditions may be experienced together. Future research should directly measure perfectionism, test anxiety, employment stress, perceived evaluation pressure, and their relationships with learning engagement before drawing conclusions about their effects.

### 4.7 Limitations

This study has several limitations. First, it was conducted at a single center, and the sample was predominantly female, held associate degrees, and came from rural backgrounds. Only seven participants were classified as having a dominant Open-Mindedness tendency. The relatively small pilot sample also limited the psychometric evaluation of the knowledge assessment, particularly the stable estimation of item-discrimination indices and dimensionality. These characteristics limit the statistical stability of subgroup comparisons and the generalizability of the findings. The dominant-trait classification was also an exploratory simplification and should not be interpreted as a definitive personality typology. Voluntary participation may additionally have introduced self-selection bias.

Second, the one-group pre-test–post-test design did not include randomization, a control group, or long-term follow-up. Consequently, the study cannot establish that the virtual simulation program caused the observed knowledge improvement. Using the same knowledge test before and after training may also have introduced testing effects. In addition, personality was assessed after the intervention, limiting temporal ordering and its interpretation as a prospective predictor.

Third, the outcome assessment focused primarily on short-term knowledge scores. The study did not determine whether the observed patterns extended to clinical competence, behavioral performance, transfer to actual practice, or patient-care outcomes. Future research should incorporate objective simulation-performance measures and longer-term assessments.

Fourth, the platform-recorded indicators, including the number of learning sessions and average simulation scores, provided relatively coarse representations of engagement. More detailed learning analytics are required to distinguish deliberate practice, feedback use, and deep cognitive engagement from mechanical repetition.

Finally, the qualitative phase purposively selected participants with relatively high knowledge gains within each personality group. Their accounts may not represent learners who obtained limited benefit, disengaged from the program, or experienced greater barriers. The one-third inclusion threshold used in constructing the consensus map may also have excluded meaningful minority experiences. Furthermore, the study did not formally test mediation, moderation, interaction, or reciprocal effects. Longitudinal and experimental studies are needed to examine the temporal and potentially causal relationships among personality, learning behaviors, emotional experiences, environmental conditions, and learning outcomes.

## 5 Conclusion

This study observed that virtual simulation training was followed by improvements in fall-management knowledge scores among standardized training nurses. The quantitative findings showed that learning behaviors, particularly the number of learning sessions and average simulation scores, were the strongest proximal correlates of post-test knowledge. None of the five continuous personality dimensions remained statistically significant after learning behaviors were included in the final regression model. However, in the secondary exploratory group comparison, the Conscientiousness group showed a greater mean knowledge gain than the Neuroticism group. This finding should be interpreted cautiously given the borderline omnibus significance, unequal subgroup sizes, and the use of dominant-trait grouping as an exploratory analytical simplification.

The qualitative findings provided complementary contextual insights. Conscientiousness tended to co-occur with self-regulated learning behaviors, including planning, repeated practice, and systematic knowledge consolidation. Neuroticism was frequently reported alongside anxiety, confusion, time pressure, and difficulties in maintaining engagement. Agreeableness was associated with supportive peer behaviors and a collaborative learning atmosphere, although group-level effects were not directly measured. Taken together, these findings provide preliminary evidence that personality characteristics may be associated with different behavioral, emotional, and interpersonal patterns of engagement rather than directly determining learning outcomes.

The findings support the use of Bandura’s triadic reciprocal determinism as a theory-informed framework for understanding the interplay among personal characteristics, learning behaviors, and the virtual simulation environment in technology-mediated clinical education. The qualitative findings further highlighted cognitive, affective, and interpersonal experiences that were not fully captured by platform-recorded indicators alone.

In practice, behavioral indicators such as practice frequency and simulation performance may be more useful than personality classification alone for identifying learners who may require additional support. Educators may consider providing clearer structure for learners experiencing anxiety, opportunities for meaningful repeated practice, and collaborative activities that encourage peer support. These strategies should respond to learners’ observed behaviors and expressed needs rather than treat personality traits as fixed labels or limitations. Future longitudinal and experimental studies are needed to examine the temporal and potentially causal relationships among personality characteristics, learning behaviors, environmental conditions, and learning outcomes in virtual simulation settings.

## Data Availability

The original contributions presented in the study are included in the article/Supplementary material, further inquiries can be directed to the corresponding author/s.
