# Supplementary material for: Personality-related learning pathways in virtual simulation: a theory-informed mixed-methods study among standardized training nurses
Source: Front Psychol. 2026 Aug 12;17:1876039. doi: 10.3389/fpsyg.2026.1876039 (PMC13506918; doi:10.3389/fpsyg.2026.1876039)
Supplement: Supplementary file 1 [file Data_Sheet_1.pdf]

Supplementary File 1

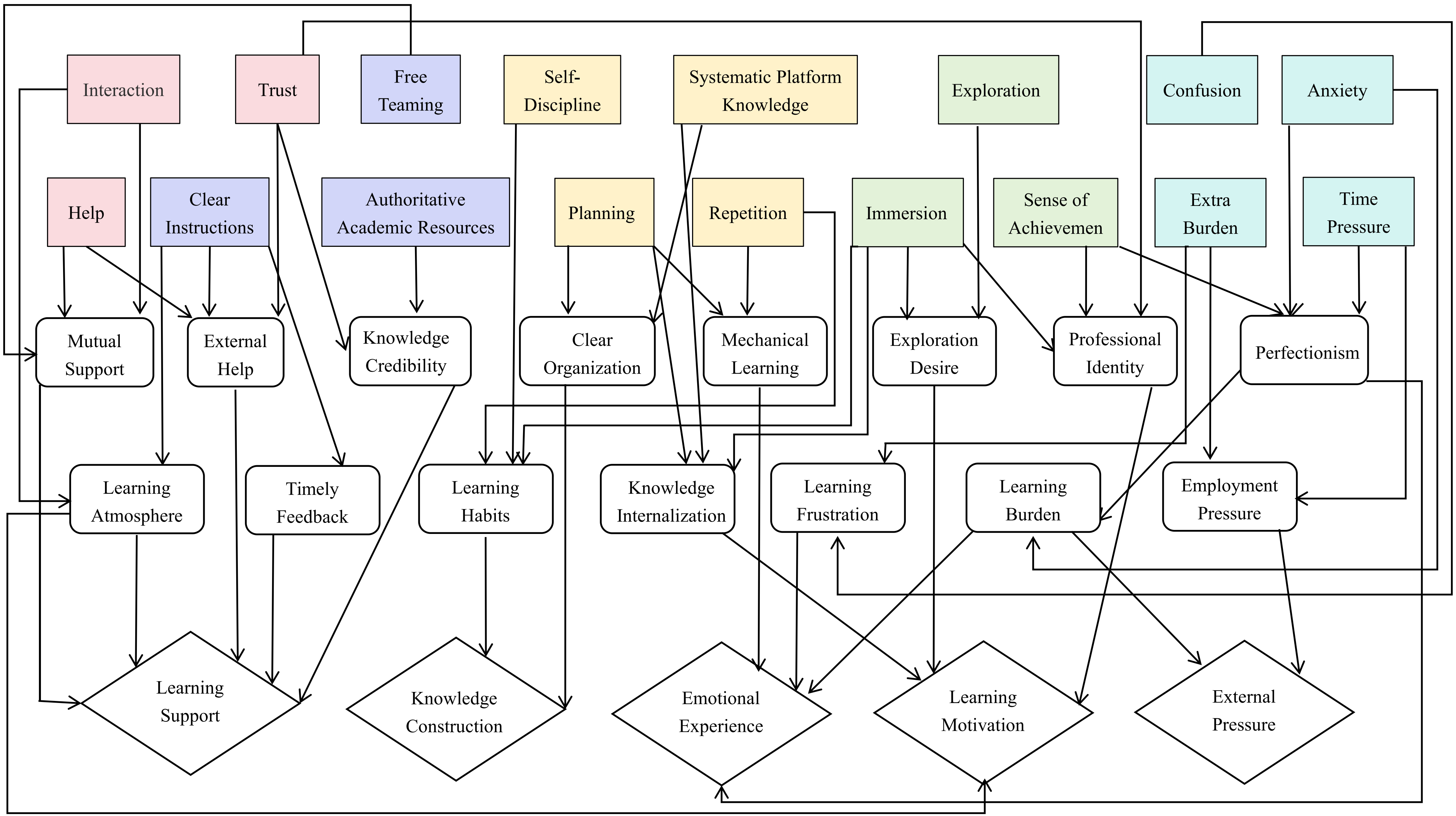

Note: Squares represent the raw constructs of resident nurses with different personality types: pink for Agreeableness, purple for Extraversion, yellow for Conscientiousness, green for Open-Mindedness, and blue for Neuroticism; ovals represent relational constructs; diamonds represent final constructs.
